# Recent insights into mechanisms preventing ectopic centromere formation

**DOI:** 10.1098/rsob.210189

**Published:** 2021-09-08

**Authors:** Qianhua Dong, Jinpu Yang, Jinxin Gao, Fei Li

**Affiliations:** Department of Biology, New York University, New York, NY 10003-6688, USA

**Keywords:** CENP-A, chromosomes segregation, ectopic centromeres, epigenetics, kinetochore, neocentromere

## Abstract

The centromere is a specialized chromosomal structure essential for chromosome segregation. Centromere dysfunction leads to chromosome segregation errors and genome instability. In most eukaryotes, centromere identity is specified epigenetically by CENP-A, a centromere-specific histone H3 variant. CENP-A replaces histone H3 in centromeres, and nucleates the assembly of the kinetochore complex. Mislocalization of CENP-A to non-centromeric regions causes ectopic assembly of CENP-A chromatin, which has a devastating impact on chromosome segregation and has been linked to a variety of human cancers. How non-centromeric regions are protected from CENP-A misincorporation in normal cells is largely unexplored. Here, we review the most recent advances on the mechanisms underlying the prevention of ectopic centromere formation, and discuss the implications in human disease.

## Graphical abstract

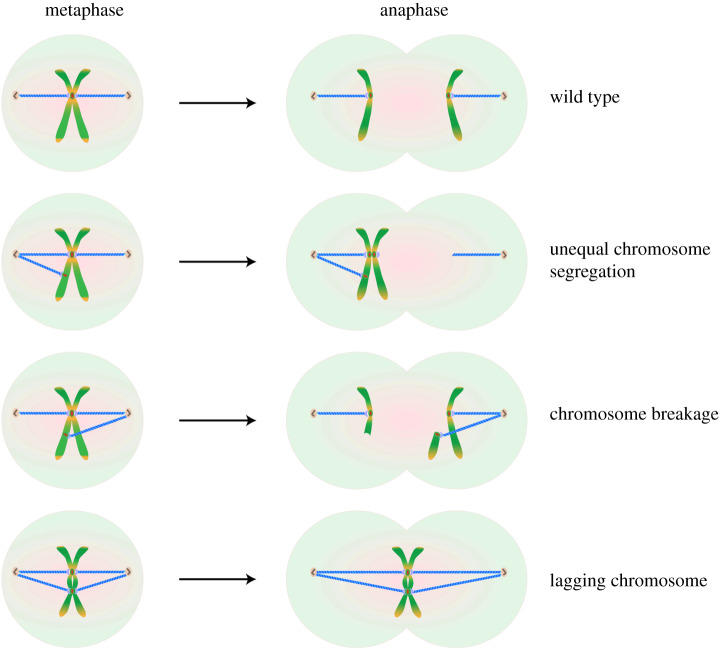

## 1. Centromeres and genome stability

**Figure 1.**
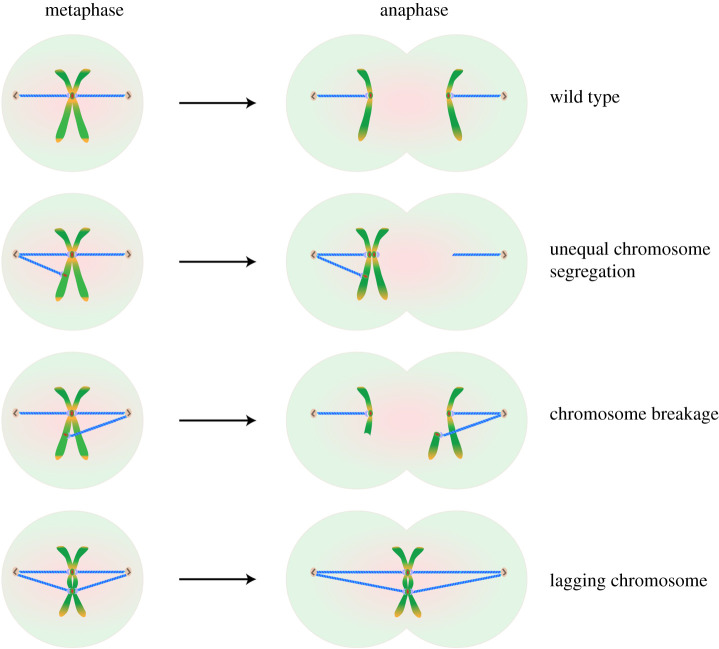
Formation of ectopic centromeres can lead to chromosome segregation errors. Normal centromeres ensure equal segregation of chromosomes into daughter nuclei. Unequal chromosome segregation, chromosome breakage and lagging chromosomes can be observed when cells fail to prevent the formation of ectopic centromeres.

During each cell division, duplicated chromosomes have to be equally segregated into daughter cells to ensure genome stability and the successful transmission of genetic information across generations. To achieve this, the spindle, a micro-machine composed of microtubules, is formed when a cell starts to divide. Spindle microtubules attach to chromosomes via the kinetochore, a large protein complex consisting of over 100 proteins, and physically pull the two chromatids of the same chromosome towards opposite poles (for reviews, see [1,2]). The centromere (see box 1, Glossary) is a region of specialized chromatin where the kinetochore assembles. This unique chromatin domain, first described by Walther Flemming in 1882 [3], provides the foundation for precise assembly of the kinetochore complex and also serves as a site for sister chromatid attachment (for reviews, see [4–6]). Centromeres thus play a crucial role in chromosome segregation during mitosis and meiosis. In most eukaryotes, each chromosome must contain a single centromere. Chromosomes without centromeres are eventually lost from cells since there are no microtubule attachments. On the other hand, chromosomes with more than one active centromere can induce chromosome breakage, unequal chromosome segregation and lagging chromosomes. Such defects often result in aneuploidy and genome instability, both of which have been recognized as hallmarks of cancers [7,8] (figure 1). Dysfunctional centromeres have thus been implicated in many human diseases, such as cancer, birth defects, infertility and ageing [4,6,9,10]. Much research has been focused on how centromeres are assembled. It is equally important, though, to understand how the formation of functional centromeres at non-centromeric regions is prevented. We review here recent advances in our understanding of the prevention of ectopic centromere formation. We will also discuss key questions and remaining challenges in the field as well as the implications of these findings in human disease.

## 2. CENP-A as an epigenetic mark for centromere identity

The most common types of centromere are the large and complex regional centromeres. Regional centromeres contain multiple microtubule attachment sites per centromere and can span a few kilobases to several megabases of DNA. DNA sequences underlying regional centromeres vary from species to species [4,11]. Neocentromeres can naturally form in regions without canonical centromere DNA sequences [12–16]. In addition, neocentromeres can also generate experimentally in many species, such as *Schizosaccharomyces pombe*, *Candida albicans*, *Drosophila melanogaster*, and chicken and human cells [17–24]. These works provide compelling evidence that regional centromeres are regulated epigenetically. Nevertheless, centromeric DNA sequences also contribute to centromere structure and function [25–27].

Box 1. Glossary.

Acentric: lacking a centromere
Aneuploidy: abnormal number of chromosomes
Centromere: a specialized chromatin region where the kinetochore is assembled
Epigenetic: relating to heritable phenotypic changes that do not involve alteration of the DNA sequence
Heterochromatin: tightly condensed chromatin region with relatively low gene transcription
Histone variant: non-canonical variant of a histone
Holocentric species: species with holocentric chromosomes, of which the entire length functions as the centromere
Kinetochore: a large multi-protein complex that interacts with microtubules to facilitate chromosome segregation
Neocentromeres: new centromeres that form at non-canonical centromeric regions
Point centromere: the centromere in budding yeast that is genetically defined by a 125 bp DNA sequence
Proteolysis: enzymatic breakdown of proteins or peptides into amino acids
Regional centromeres: large and complex centromeres that form multiple microtubule attachment sites per chromosome

The conserved centromere-specific histone H3 variant, CENP-A, plays an essential role in centromere identity by acting as a key epigenetic mark for centromeres. CENP-A was first identified as a human autoantigen in human scleroderma patients, and was subsequently found to copurify with histones and nucleosomes [28–30]. The histone variant, together with histones H4, H2A and H2B, is assembled into unique CENP-A nucleosomes, interspersed with the canonical histone H3 within centromeres [31–34]. Post-translational modifications of the canonical histone H3 at centromeres are important for the proper assembly of CENP-A chromatin [35,36]. The CENP-A nucleosome directly interacts with the kinetochore subcomplex, CCAN (constitutive centromere-associated network), which is known to function as a platform to recruit outer kinetochore proteins [5,37–40]. Overexpression of CENP-A^CID^ in *Drosophila* results in mislocalization of CENP-A in non-centromeric regions, which promotes ectopic kinetochore assembly [41,42]. Artificially tethering CENP-A^CID^ using lacO/LacI system to chromosome arms in *Drosophila* cells leads to the formation of functional centromeres. The ectopic CENP-A^CID^ chromatin can self-propagate even without lacO/LacI system [43]. Mislocalized CENP-A in fission yeast and humans also can recruit kinetochore components [34,44–46]. Neocentromeres that form at non-centromeric chromatin also always contain CENP-A. The presence of CENP-A nucleosomes in chromatin thus defines centromere identity.

CENP-A proteins across species share a conserved C-terminal histone fold domain (HFD), which carries the CENP-A targeting domain (CATD). CATD containing the first loop (L1) and the second α-helix (α2) is necessary and sufficient for centromeric localization of CENP-A in vertebrates [47,48]. On the other hand, the N terminus domain of CENP-A is extremely divergent in length and sequence [4,49]. It has been suggested that CENP-A evolves adaptively in concert with the underlying centromeric sequence [50]. Loading of CENP-A to centromeres is regulated by the conserved CENP-A-specific chaperone, HJURP/Scm3, in vertebrates and yeasts [51–56]. HJURP is recruited to centromeres by the Mis18 complex, which acts as the licensing factor for centromere deposition [57–59]. Mis18 directly interacts with CENP-C, a CCAN subunit [60,61]. CAL1 is the functional orthologue of HJURP/Scm3 in *Drosophila* that guides the deposition of CENP-A [62,63]. Interestingly, CAL1 also directly binds CENP-C, suggesting that it may have similar functions to the Mis18 complex [63,64]. Several other factors have also been identified to function in CENP-A assembly (see reviews [3,4,65]). The timing of CENP-A loading through the cell cycle varied between organisms and cell types. CENP-A is deposited at early G1 in human cells and *Drosophila* somatic tissues [66–68]. However, CENP-A is loaded at G2 in fission yeast and plant tissues [69–71]. The transcription of CENP-A is also cell cycle-regulated [72–78].

Another notable feature of regional centromeres is that they are often embedded in epigenetically distinct heterochromatin. The gene-poor chromatin domain is transcriptionally silenced, enriched with the histone H3K9 methylation. Although heterochromatin *per se* appears antithetical to CENP-A chromatin [79,80], the silenced chromatin domain surrounding centromeres can also create a favourable chromatin environment for CENP-A incorporation and contributes to the centromere assembly [17,42,81–85]. Neocentromeres also often emerge from regions near heterochromatin in *S. pombe* and *Drosophila* [17,19,41,42,84]. Nevertheless, human neocentromeres were found at both heterochromatic and nonheterochromatic regions [86,87]. In chicken DT40 cells, neocentromeres can also form at both transcriptionally active and silenced sites [20]. Interestingly, a recent 4C (circularized chromosome conformation capture) analysis of chicken DT40 cells containing neocentromeres that are not localized near heterochromatin revealed that these neocentromeres generally associate with specific heterochromatic regions in the 3D organization through long-range contacts [88]. Removing H3K9 trimethylation in human artificial chromosomes also causes a reduction of CENP-A in centromeres and mitotic missegregation [89]. The exact role of heterochromatin in centromere assembly still needs to be further explored.

In contrast with regional centromeres, some budding yeasts, such as *Saccharomyces cerevisiae*, contain genetically defined ‘point centromeres'. The point centromere in budding yeast is 125 bp long, forming a single microtubule attachment site. The DNA sequence is necessary and sufficient for centromere formation [50,90,91]. Unlike regional centromeres, point centromeres lack pericentromeric heterochromatin. The single centromere nucleosome in the point centromere also contains the CENP-A homologue, Cse4, which is crucial for kinetochore assembly and chromosome segregation [92–94]. Another type of centromere is observed in holocentric species, where use the entire chromosome as the centromere. Holocentric chromosomes are found in worms, insects and some plant species [95]. A genome-wide mapping study by ChIP-chip in *Caenorhabditis elegans* demonstrated that CENP-A is incorporated at low density in non-repeated regions across approximately half of the genome [96]. Nevertheless, CENP-A was lost in some holocentric insects [97].

## 3. Mislocalization of CENP-A has deleterious effect on chromosome segregation

Overexpressed CENP-A^CID^ in *Drosophila* induces ectopic assembly of CENP-A^CID^ chromatin at non-centromeric regions. Mislocalized CENP-A^CID^ chromatin can promote the formation of functional kinetochores. As a result, cells exhibit mitotic delays, anaphase bridges, chromosome fragmentation and spindle disorganization, which lead to cell and organism lethality [41]. Similarly, overexpression of CENP-A^Cnp1^ in fission yeast also results in the assembly of CENP-A^Cnp1^ chromatin at non-centromeric regions. The mispositioned CENP-A^Cnp1^ is able to recruit kinetochore proteins. Consequently, fission yeast cells overexpressing CENP-A^Cnp1^ also display chromosome missegregation during mitosis and meiosis [44,98]. Overexpression of CENP-A in human cells also results in recruitment of a subset of kinetochore proteins to non-centromeric sites, but is not sufficient to assemble functional ectopic kinetochores. These cells exhibit lagging chromosome, micronuclei formation and abnormal mitotic exit [34,45,46]. It has been shown that overexpression of CENP-A in human cells causes reduced kinetochores in native centromeres and unstable kinetochore–microtubule interaction, which may contribute to the cellular defects in these cells [46,99]. Overexpression of CENP-A also leads to misregulation of gene expression, which can also contribute to the defects [99–101]. Interestingly, a recent study revealed that the impact of CENP-A overexpression on cell fate in human cell lines is dependent on the tumour suppressor p53. When p53 is functional, overexpression of CENP-A promotes cell cycle arrest, senescence and radiosensitivity. However, when p53 is inactivated, CENP-A overexpression instead promotes epithelial–mesenchymal transition. The cell fate changes in these cells probably result from transcriptional reprogramming induced by CENP-A overexpression [101]. Improper incorporation of CENP-A thus has a detrimental impact on cells. To preserve genome integrity and cell viability, cells have to be equipped with robust prevention mechanisms to eliminate the erroneously localized CENP-A.

It has been shown that in a variety of organisms, excess CENP-A preferentially targets the region near heterochromatin [42,44,50,98,102]. On the other hand, CENP-A can also be targeted to highly accessible chromatin regions, where histone turnover occurs, such as DNase I hypersensitive sites and transcription factor binding sites [102–104]. It was proposed that CENP-A may be opportunistic and can be incorporated into these regions in competition with histone H3 [102]. In fact, CENP-A can occasionally mistarget to non-centromeric regions even when expressed at a normal level [34,104,105]. This process can be, at times, beneficial for cell survival. For example, opportunistically mislocalized CENP-A may provide the seeding site for the formation of neocentromeres on acentric chromosomes generated during aberrant mitosis, which can play an important role in genome evolution and speciation [9,13,49].

## 4. CENP-A mislocalization and cancers

Upregulation of CENP-A expression has been found in more than 20 different human tumours. CENP-A overexpression has thus been recommended for use as a biomarker of poor patient prognosis and as a predictive biomarker for chemotherapy [10,106–112]. Knockdown of overexpressed CENP-A in hepatocellular carcinoma can inhibit tumour growth [113]. Elevated expression of HJURP has also been found in a variety of cancers [10,106,114]. A recent study reported that the tumour suppressor p53 binds the promoters of CENP-A and HJURP to repress their transcription. Both CENP-A and HJURP are upregulated following the loss of p53 [115]. This may explain why CENP-A and HJURP are highly expressed in cancer cells.

In cancer cells with elevated CENP-A level, CENP-A-containing nucleosomes are assembled at non-centromeric regions. Similar to yeasts and flies, overexpressed CENP-A in human cancer cells can result in mislocalization to both highly accessible open chromatin regions and heterochromatic loci, such as subtelomeres [103]. Ectopic assembly of CENP-A chromatin in cancer cells depends on the DAXX, a histone H3.3 chaperone unique to metazoans [100]. DAXX deletion can suppress chromosome segregation defects in cells overexpressing CENP-A [46]. Increased ectopic CENP-A directly correlates with mitotic defects [46,116]. As mentioned above, overexpressed CENP-A can weaken the endogenous kinetochore [46,99], which may lead to chromosome missegregation defects observed in CENP-A-overexpressing cancer cells. CENP-A at non-centromeric chromatin can also interfere with normal transcriptional regulation. For example, it has been shown that many genes implicated in apoptosis, cell cycle regulation, centromere and kinetochore functions are misregulated in a wide spectrum of cancer types with high CENP-A level [106,112,113]. Overexpression of CENP-A can lead to broad and rapid changes in gene expression across the genome [99–101]. CENP-A mislocalization can also promote amplification of nearby genes and lead to overexpression of the key oncogene, *MYC* [103]. How CENP-A overexpression drives cancer progression remains to be determined.

## 5. Ubiquitin-mediated proteolysis prevents ectopic localization of CENP-A

**Figure 2.**
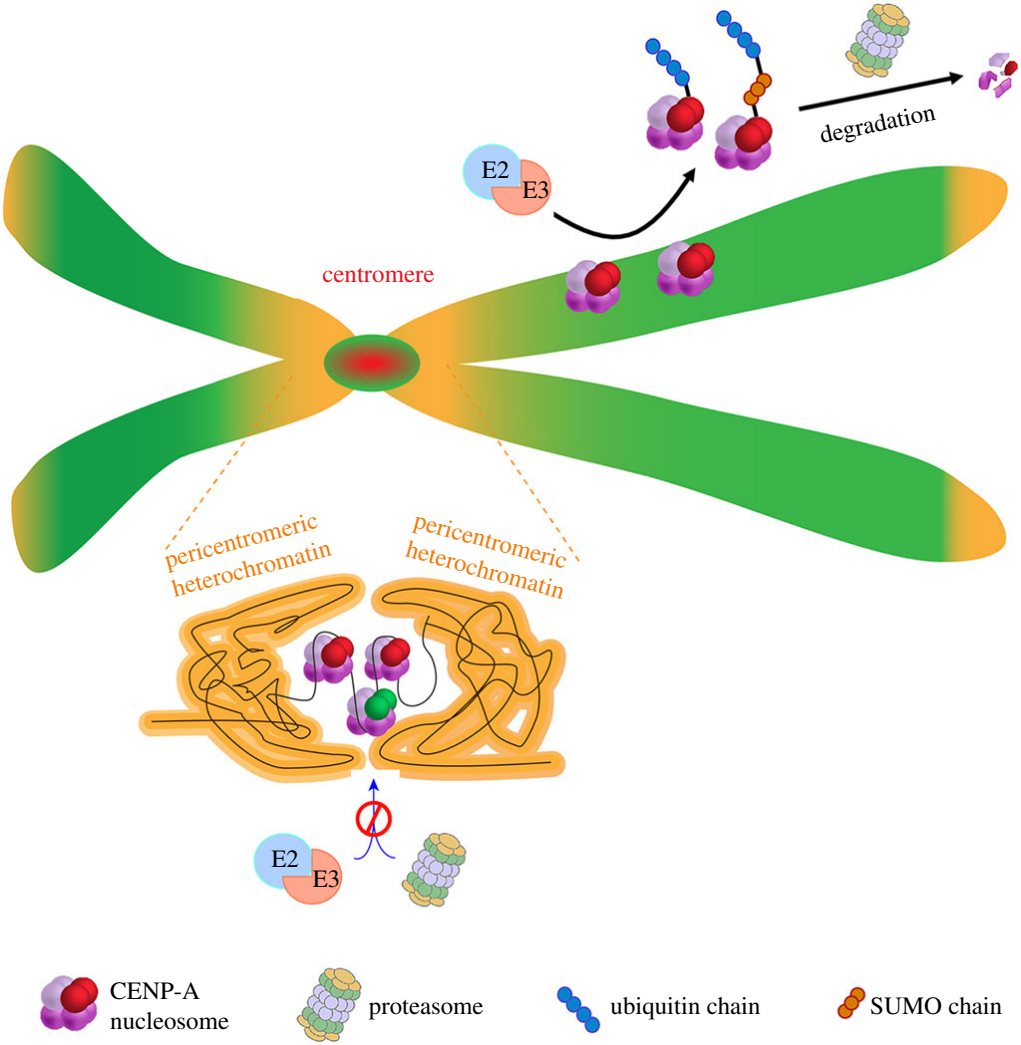
Ubiquitin-mediated proteolysis of CENP-A prevents ectopic CENP-A assembly. Ubiquitin-mediated CENP-A degradation is a conserved mechanism used for inhibiting misincorporation of CENP-A to chromosome arms. Sumoylation of CENP-A can promote ubiquitin-mediated proteolysis of CENP-A. The illustration also shows that pericentromeric heterochromatin forms a distinct high-order structure that protects centromeric CENP-A from ubiquitin-mediated degradation. E2, E2 ubiquitin-conjugating enzyme. E3, E3 ubiquitin ligase.

One of the best-studied mechanisms involved in the prevention of CENP-A mispositioning is ubiquitin-dependent proteolysis of CENP-A (figure 2). The role of ubiquitin-mediated proteolysis in restricting CENP-A to centromeres was first found in budding yeast [117]. A ubiquitin E3 ligase, Psh1, subsequently was identified to mediate CENP-A^Cse4^ degradation by targeting its CATD domain [118,119]. Its N-terminal domain has been shown to be important for CENP-A^Cse4^ proteolysis [120]. A number of ubiquitin E3 ligase components, including Ubr1 [121], Doa1 (WD-repeat protein) [120], F-box proteins Rcy1 [122], Met30 and Cdc4 [123], have also been identified to mediate the level of CENP-A^Cse4^ to prevent its mistargeting. A ubiquitin protease, Ubp8, is able to deubiquitylate CENP-A^Cse4^, and functions in concert with Psh1 to mediate the CENP-A^Cse4^ proteolysis [124]. The interaction between CENP-A^Cse4^ and Psh1 requires a proline isomerase, Fpr3. It is proposed that the structural change between the *cis* and *trans* form of CENP-A^Cse4^ regulated by Fpr3 may be important for the CENP-A^Cse4^ degradation by Psh1 [125]. A recent study [126] also showed that the Dbf4-dependent kinase complex (DDK), well studied for its role in the regulation of DNA replication initiation, mediates Psh1-dependent CENP-A^Cse4^ proteolysis. The study further showed that the role of DDK in CENP-A^Cse4^ proteolysis is independent of its role in DNA replication [126].

Small ubiquitin-like modifier (or SUMO) proteins are a family of small proteins similar to ubiquitin that is covalently attached to other proteins. Sumoylation is involved in many cellular processes, including ubiquitination. The SUMO-targeted ubiquitin ligase (STUbL) links SUMO and ubiquitin modification pathways to facilitate proteolysis of cellular substrates [127]. Sumoylation of CENP-A^Cse4^ also acts as an important regulator of ubiquitin-mediated proteolysis of CENP-A^Cse4^. CENP-A^Cse4^ is sumoylated by E3 SUMO ligases Siz1 and Siz2. The STUbL protein Slx5 regulates ubiquitin-mediated proteolysis of CENP-A^Cse4^ to prevent mislocalization of the H3 variant by targeting lysine 65 (K65) in CENP-A^Cse4^. However, Slx5-mediated CENP-A^Cse4^ proteolysis acts in a manner that is independent of Psh1 [128,129]. Interestingly, histone H4 was also implicated in SUMO/ubiquitin-mediated proteolysis of CENP-A^Cse4^ in budding yeast [130,131].

Ubiquitin-mediated CENP-A degradation has also been shown in fission yeast and *Drosophila* to prevent the formation of ectopic CENP-A chromatin [44,132,133]. In *Drosophila*, the F-box protein Ppa, the ubiquitin E3 ligase APC/C and the E3 ligase CUL3/RDX have been found to regulate the proteolysis of the CENP-A^CID^ [134–136]. A recent study further showed that serine 20 (S20) of CENP-A^CID^ is phosphorylated by casein kinase II. The phosphorylation regulates the stability of prenucleosomal CENP-A^CID^ via the Ppa-proteasome pathway and promotes the removal of CENP-A^CID^ from ectopic sites [137].

Ubiquitin-dependent proteolysis of CENP-A has also been used in vertebrates to regulate centromere functions. In human cells, the herpes simplex virus type 1 protein ICP0 has been shown to be able to induce the proteasome-dependent degradation of CENP-A [138]. Ubiquitin-mediated CENP-A degradation was also implicated in senescent human cells [139]. A recent genetic screen in human cell lines identified several factors in the SUMO/ubiquitin pathway that affect centromere maintenance, including a CUL3-RING ubiquitin ligase component, KEAP1, the ubiquitin-conjugating enzyme E2 A (UBE2A) and the SUMO-specific peptidase 6 (SENP6). This study showed that SENP6 is also involved in the loading of new CENP-A at centromeres [140]. The role of SENP6 in centromere function was also found by several other parallel studies [141–143]. Nevertheless, CENP-A does not appear to be the direct target of SENP6 [140–143]. In addition, a human ubiquitin isopeptidase, USP48, is implicated in centromere function [143]. These data suggest that ubiquitin-mediated CENP-A proteolysis is a common mechanism used to prevent ectopic assembly of CENP-A chromatin across species. Nevertheless, ubiquitin-independent CENP-A proteolysis has also been suggested to contribute to CENP-A stability and centromere integrity [117,131].

Overexpression of CENP-A^Cse4^ is lethal in budding yeast cells lacking Psh1 [118,119], but has little impact on growth in wild-type cells [117,144]. By contrast, overexpression of CENP-A in wild-type organisms with regional centromeres, such as fission yeast and *Drosophila,* causes severe chromosome missegregation and growth defects [41,44,46]. One possible scenario is that the point centromere of budding yeast might need much less CENP-A^Cse4^ compared to other species which contain large regional centromeres; CENP-A^Cse4^ may thus be subject to more efficient ubiquitination, leading to its rapid degradation [132]. Another non-mutually exclusive explanation is that it may be due to the epigenetic nature of CENP-A in regional centromeres [41,43,44]. The sequence-dependent CENP-A^Cse4^ chromatin at ectopic regions in budding yeast may be less stable, which can be quickly removed during the cell cycle.

### 5.1. How is CENP-A at centromeres protected from degradation?

These exciting discoveries also raise another important question: how is CENP-A at centromeres protected from ubiquitin-mediated degradation? HJURP/Scm3, the CENP-A chaperone, in human cells and *S. cerevisiae* has been suggested to associate with CENP-A to inhibit its degradation [51,118]. CAL1, the counterpart of HJURP/Scm3 in *Drosophila*, was also implicated in protecting CENP-A^CID^ from degradation [63,135]. Additionally, kinetochore proteins may be involved in this process [117,145]. A recent study has shown that pericentromeric heterochromatin in fission yeast also plays a crucial role in protecting CENP-A^Cnp1^ from ubiquitin-mediated degradation [132]. The higher-order architecture of heterochromatin may create a protective environment for CENP-A to assemble into a stable domain that can be propagated across generations (figure 2). By contrast, CENP-A promiscuously incorporated to the highly accessible regions may be subject to a high rate of turnover, and less likely to form stable CENP-A chromatin. The heterochromatin-mediated CENP-A protection mechanism may provide a partial explanation of why regional centromeres often are flanked with heterochromatin. Interestingly, loss of heterochromatin in human cells also results in reduction of CENP-A in centromeres, although the exact mechanism is still unknown [89]. Defects in heterochromatin are linked to tumorigenesis [146–149], and its role in centromere function may be a contributing factor. Nevertheless, the loss of heterochromatin only results in partial degradation of CENP-A^Cnp1^ in fission yeast, indicating that additional pathways also participate in the process [132].

## 6. Cell-cycle regulation of CENP-A transcription as a key control mechanism for CENP-A level

Proper centromere assembly depends on the tight regulation of the CENP-A level. CENP-A level is subject to not only post-translational regulation, but also transcriptional control. Reduction of CENP-A RNAs causes lagging chromosomes [139,150]. A recent study found that the cyclin-dependent kinase 5 regulatory subunit-associated protein 2 (Cdk5rap2) functions as a transcriptional activator of CENP-A in human cells [151]. Cdk5rap2 binds the CENP-A promoter and upregulates CENP-A transcription. Deletion of Cdk5rap2 results in a reduced expression level of CENP-A RNA. The study further showed that transcriptional regulation of CENP-A by Cdk5rap2 partially contributes to the chromosome segregation defects observed in the Cdk5rap2 knockdown cells [151].

**Figure 3.**
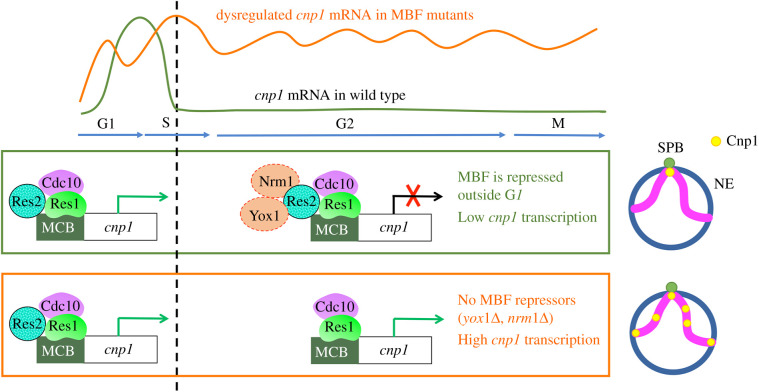
Cell cycle-regulated CENP-A transcription as a key step to control CENP-A level. Model for cell cycle-regulated CENP-A^Cnp1^ transcription by the MBF complex. During the G1/S transition, the MBF core complex consisting of Cdc10, Res1 and Res2 binds to the MCB motif within the CENP-A^Cnp1^ promoter to activate CENP-A^Cnp1^ transcription. The MBF core complex also activates the transcription of the *yox1* and *nrm1* repressor genes, as well as other genes involved in DNA replication. Yox1 and Nrm1 subsequently bind to the MBF core complex via interaction with Res2, leading to the inhibition of the transcriptional induction activity of the complex. This establishes a negative feedback loop preventing the constitutive activation of CENP-A^Cnp1^ for the rest of the cell cycle. Without Nrm1 and Yox1, the MBF activator core complex remains active throughout the cell cycle, resulting in an abnormal accumulation of CENP-A^Cnp1^ transcripts. If the level of CENP-A^Cnp1^ exceeds a certain threshold, the cell starts displaying CENP-A^Cnp1^ mislocalization and consequently mitotic defects. MBF, MluI box-binding factors; MCB, MluI cell cycle box; SPB, spindle pole body; NE, nuclear envelope.

CENP-A transcription is usually cell cycle-regulated, which appears to be a universal feature across eukaryotes. Interestingly, CENP-A transcription is generally uncoupled from canonical histone transcription [72–78]. The molecular mechanism underlying the temporal control of CENP-A transcription remains elusive. Using a genetic screen, Aristizabal-Corrales *et al*. [152] identified the MBF (MluI box-binding factors) complex as a key regulator of temporal control of CENP-A^Cnp1^ transcription in fission yeast. CENP-A^Cnp1^ transcription in fission yeast occurs at G1, leading to an almost twofold increase in CENP-A^Cnp1^ during the S-phase [74,152]. The MBF complex is a major transcription factor regulating the transcription of genes required for DNA replication during the G1/S transition of the cell cycle [153,154]. The core of the MBF complex consists of Res2, a DNA-binding protein, Res1 and Cdc10, and is involved in both the transcriptional activation and repression activities [155–158]. Nrm1 and Yox1 are negative regulators of the MBF complex [159,160]. Aristizabal-Corrales *et al.* demonstrated that the periodic transcription of CENP-A^Cnp1^ is lost in MBF mutants, resulting in the increased level of CENP-A^Cnp1^ and consequently CENP-A mislocalization to non-centromeric regions and mitotic defects (figure 3). They further showed that MBF binds the MCB (MluI cell cycle box) motif in the CENP-A^Cnp1^ promoter to restrict CENP-A^Cnp1^ transcription to G1. Mutations of the MCB motif cause constitutive CENP-A^Cnp1^ expression and chromosome missegregation [152]. The MBF complex is functionally analogous to the E2F complex in plants and metazoans, which is known to be a key transcription factor for controlling the G1/S and also G2/M [154,161]. In *Arabidopsis*, E2fa binds to the promoter of CENP-A *in vivo* and is important for CENP-A transcription [77]. Downregulation of E2F/RBR in human cells also results in increased RNA and protein levels of CENP-A [162]. These works suggest that cell cycle-regulated CENP-A transcription is another key step in centromere regulation to ensure an optimal level of CENP-A is generated.

## 7. Removing ectopic CENP-A via a DNA replication-driven mechanism

A recent exciting finding suggested that DNA replication may act as a key player in preventing the mislocalization of CENP-A [163]. In human cells, CENP-A assembles in centromeres containing almost identical α-satellite DNA repeats, making it difficult to detect the precise binding sites of the histone variant. New studies recently enabled researchers to generate centromere reference models for 23 human chromosomes [164,165]. Using these reference models, Nechemia-Arbely *et al*. [163] examined how DNA replication affects CENP-A distribution in human cells. The authors found that the positions of almost all CENP-A nucleosomes in centromeres were retained before and after DNA replication. In addition, Nechemia-Arbely *et al*. showed that CENP-As also incorporate into non-centromeric chromosome arms during the G1 phase [163]. Remarkably, the ectopic CENP-A peaks found in G1-phase cells were not detected in G2-phase cells, indicating that mislocalized CENP-A at non-centromeric regions was removed after DNA replication. They further showed that more than 90% of non-centromeric CENP-A-binding sites were replicated in early or mid-S phase, and the mislocalized CENP-A was removed immediately after DNA replication. By contrast, the centromeric DNA sequence is replicated in late S-phase, and CENP-As in centromeres were found at the same positions before and after DNA replication [163].

**Figure 4.**
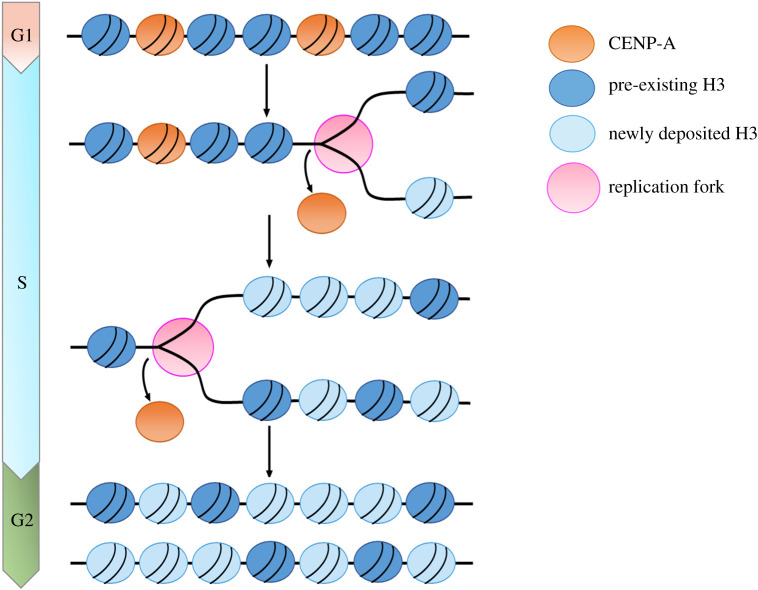
Ectopic CENP-A elimination during DNA replication. CENP-A incorporates into non-centromeric chromosome arms during the G1 phase. During the S-phase, CENP-A-containing nucleosomes in chromosome arms are evicted by the replication fork. However, the ectopic CENP-As are not re-deposited into chromatin after DNA replication.

These data strongly support the idea that all the pre-existing nucleosomes including CENP-A-containing nucleosomes in chromosome arms are evicted during S-phase by the replication fork; however, CENP-A nucleosomes at non-centromeric regions are not re-incorporated into chromatin after DNA replication (figure 4), whereas CENP-A in centromeres is retained after eviction by the replication fork. The authors further showed that the CCAN complex appears to be still associated with centromeric CENP-A during DNA replication and that CENP-C, a key component of CCAN, plays an essential role in the maintenance of centromeric CENP-A during S-phase [163]. Together, this work suggested that DNA replication can function as an error correction mechanism to ensure centromere integrity by removing ectopically localized CENP-A. Exactly how mislocalized CENP-A is removed by the DNA replication process remains unclear. Interestingly, a recent study showed that the F-box protein Ppa in *Drosophila* regulates CENP-A^CID^ level in S-phase [135]. It is possible that the CENP-A nucleosomes at non-centromeric regions after eviction by the replication fork are degraded via ubiquitin-mediated proteolysis during DNA replication. In addition, neocentromeres form at non-centromeric chromosome arms [12,13]. It is still unknown how neocentromeres escape from the clearance by the DNA replication-mediated error correction mechanism.

## 8. Other factors preventing mislocalization of CENP-A

### 8.1. Chromatin remodelling factors

Chromatin remodellers are multi-protein complexes that regulate chromatin accessibility and nucleosome positioning by using energy from ATP hydrolysis. Multiple chromatin remodelling factors, including the CHD remodelling protein Hrp1, and the RSF and Ino80 complexes, have been implicated in CENP-A chromatin assembly [166–169]. Emerging evidence also links chromatin remodelling factors to the prevention of ectopic CENP-A chromatin assembly.

#### 8.1.1. Facilitates chromatin transcription/transactions

The FACT (facilitates chromatin transcription/transactions) complex is a conserved chromatin remodelling complex, composed of two subunits, Spt16 and SSRP1 (Pob3 in yeast). The complex is responsible for recycling dissociated histones during transcription elongation when nucleosomes are transiently disassembled in front of RNA polymerase II. Dysfunctional FACT results in the loss of histone H3 nucleosome across the genome in *S. cerevisiae* [170,171]. In fission yeast, FACT mutants also exhibited widespread CENP-A^Cnp1^ misincorporation in non-centromeric regions. It was proposed that CENP-A^Cnp1^ chromatin prefers to assemble de novo in the regions where the integrity of H3 chromatin is compromised [102]. However, a more recent study showed that the FACT complex may play a more direct role in preventing CENP-A^Cse4^ misincorporation in *S. cerevisiae*. The ubiquitin E3 ligase Psh1 interacts with CENP-A^Cse4^ through the CATD motif. The motif is buried within the nucleosome core, blocking the access of Psh1 to CENP-A^Cse4^ assembled into nucleosomes. The Spt16 subunit of FACT can bind to Psh1 to facilitate the interaction of Psh1 with nucleosomal CENP-A^Cse4^, which leads to CENP-A^Cse4^ degradation [172]. Interestingly, in chicken DT-40 cells and *Drosophila*, FACT has also been shown to be required for CENP-A^CID^ assembly at centromeres [173–175].

#### 8.1.2. HIR/HIRA

The HIR/HIRA complex is a conserved histone chaperone that facilitates the deposition of the histone variant H3.3 into chromatin in a replication-independent manner [176]. CAF-1, on the other hand, is a histone chaperone that promotes nucleosome formation behind the replication fork during DNA replication [177]. In budding yeast, deletion of both HIR1 (a subunit of the HIR complex) and CAF-1 results in erroneous accumulation of CENP-A^Cse4^ at euchromatin corresponding to the sites of rapidly exchanging nucleosomes in wild-type cells. It was thus proposed that CENP-A^Cse4^ mislocalization primarily results from decreased eviction of CENP-A^Cse4^ at euchromatic regions mediated by both HIR and CAF-1 [178]. However, Gerton's group [179] has found that when overexpressed, genome-wide CENP-A^Cse4^ assembly is drastically diminished in the absence of CAF-1 in budding yeast. This opposite effect led them to propose that CAF-1 interacts with CENP-A^Cse4^ to facilitate the deposition of CENP-A^Cse4^ to non-centromeric regions [179]. Further studies are needed to reconcile the discrepancy. Nevertheless, another recent study [180] showed that CENP-A^Cse4^ is mispositioned in euchromatin in the mutant of HIR2, another subunit of the HIR complex, when CENP-A^Cse4^ is overexpressed. The study further showed that HIR2 mediates CENP-A^Cse4^ proteolysis by facilitating the interaction of CENP-A^Cse4^ with Psh1 [180]. Thus, the HIRA complex may play an important role in preventing CENP-A^Cse4^ mislocalization through regulation of ubiquitin-mediated CENP-A^Cse4^ degradation.

#### 8.1.3. SWI/SNF

The SWI/SNF complex is a large, multi-subunit chromatin remodelling complex that plays important roles in the regulation of transcription, the cell cycle, and DNA repair and replication. The conserved complex uses the energy of ATP hydrolysis to mobilize and restructure nucleosomes [181]. In budding yeast, CENP-A^Cse4^ has been found to associate with non-centromeric regions in a mutant of Snf2, the catalytic subunit of SWI/SNF. But the overall level of CENP-A^Cse4^ does not change in the mutant, suggesting that the abnormal distribution of CENP-A^Cse4^ in the mutant is not due to defects in the regulation of CENP-A^Cse4^ protein level [182]. It has been further shown that the SWI/SNF complex is capable of destabilizing chromatin that contains CENP-A^Cse4^, but not H3 nucleosome *in vitro* [182]. This study suggests that the SWI/SNF complex restricts CENP-A^Cse4^ to centromeres by removing it from ectopic sites. It is unclear why SWI/SNF prefers to destabilize CENP-A^Cse4^ nucleosomes. It is speculated that the CENP-A^Cse4^ nucleosomes may be inherently less stable than H3 nucleosomes [182].

#### 8.1.4. Chromatin accessibility complex

The chromatin accessibility complex (CHRAC) is an ISWI-containing chromatin remodelling complex found in *Drosophila* and human cells. The HFD-containing protein, CHRAC14, is a subunit of the CHRAC complex. CHRAC14 is important for DNA damage response in *Drosophila*. In the absence of CHRAC14, DNA damage is unable to be efficiently repaired [183]. Notably, CENP-A^CID^ is mislocalized to damage DNA sites in the *Chrac14* mutant, resulting in the formation of ectopic kinetochore and genome instability. CHRAC14 also interacts with CENP-A^CID^ [183]. It has been shown that the induction of a double-strand break is sufficient to recruit CENP-A to the damage site [184]. It is still unclear whether the mislocalization of CENP-A^CID^ observed in the *Chrac14* mutant results from DNA repair defects in the mutant, or whether CHRAC14 plays a direct role in preventing the assembly of ectopic centromeres.

### 8.2. The NAP family protein, Ccp1

The Nucleosome Assembly Protein (NAP) family proteins are found in many organisms, and often act as histone chaperones, serving a crucial role in the assembly and disassembly of nucleosomes [185]. Using a visual genetic screen, Dong *et al*. [186] identified a NAP family protein, Ccp1, that prevents mislocalization of CENP-A^Cnp1^ in fission yeast. CENP-A^Cnp1^-GFP forms ectopic foci in the *ccp1Δ* mutants, whereas overexpression of Ccp1 results in removal of mistargeted CENP-A^Cnp1^-GFP. Ccp1 preferentially associates with CENP-A^Cnp1^ both *in vitro* and *in vivo*. Crystal structural analyses indicated that, like other NAP family proteins, Ccp1 forms a homodimeric ‘headphone’ architecture with a pronounced cleft at the centre of the dimer. The dimerization of Ccp1 is required for its anti-CENP-A loading activity [186]. But how Ccp1 actively removes CENP-A^Cnp1^ from the chromatin is not clear.

Interestingly, Ccp1 also plays an important role in modulating epigenetic stability in centromeres [186,187]. CENP-A only partially replaces the canonical histone H3 in centromeres; the centromere-specific CENP-A nucleosomes are interspersed with the histone H3 nucleosomes [5,33]. How the balance of CENP-A and H3 at centromeres is achieved is unknown. The work by Dong *et al*. [186] demonstrated that Ccp1 also appears to antagonize the CENP-A^Cnp1^ loading within centromeres. Thus, both CENP-A loading and anti-loading factors are recruited to centromeres, which may explain how CENP-A and histone H3 levels are properly maintained in centromeres. Consistent with this idea, both Ccp1 and the CENP-A chaperone Scm3 are recruited to centromeres at the end of mitosis, and dissociated from centromeres in metaphase [186]. Why Ccp1 is absent during mitosis is still unknown. The amino acid sequence of Ccp1 shows 27% identity with Vps75 in budding yeast, although Vps75 does not appear to be involved in centromere chromatin function.

### 8.3. The histone variant, H2A.Z

H2A.Z is a conserved variant of the core histone H2A, which shares about 60% sequence similarity. H2A.Z nucleosome occupancy has been implicated in both transcription activation and repression [188]. H2A.Z is also implicated in the prevention of promiscuous formation of CENP-A chromatin. In fission yeast, artificial deletion of centromeres results in the formation of neocentromeres at regions devoid of H2A.Z/Pht1 [189]. Consistent with this, CENP-A^Cnp1^-GFP in the *pht1* deletion mutant is mislocalized to non-centromeric regions [186]. It appears that Pht1 inhibits the efficient association of Scm3, which is also important for ectopic assembly of CENP-A^Cnp1^ chromatin, with the chromosome arms [189]. H2A.Z also physically associates with Ccp1. The double *pht1 ccp1* mutant shows synthetic defects in CENP-A^Cnp1^ distribution, suggesting that H2A.Z corroborates with Ccp1 to mediate the prevention of CENP-A mislocalization [186]. A recent study suggested that Pht1 in budding yeast also helps to prevent the formation of ectopic CENP-A^Cse4^ chromatin [190].

### 8.4. CENP-C

CENP-C is a key subunit of the inner kinetochore complex, CCAN. CENP-C associates with CENP-A and contributes to the stability of CENP-A nucleosomes [6,191]. A recent study also implicated CENP-C in preventing the formation of de novo centromeres. Using a genetic screen, Suma *et al*. [192] identified a mutant (*cnp3-1*) of a gene encoding the homologue of the mammalian CENP-C in fission yeast. The mutant became temperature-sensitive when CENP-A^Cnp1^ was overexpressed. The authors further showed that the *cnp3-1* mutant was prone to promiscuous accumulation of CENP-A^Cnp1^ at non-centromeric regions at the restrictive temperature when overexpressing CENP-A^Cnp1^ [192]. This work suggests that CENP-C may also play a role in restricting CENP-A^Cnp1^ at centromeres by preventing mislocalization of CENP-A^Cnp1^ to ectopic sites. But how *cnp3-1* causes misincorporation of CENP-A^Cnp1^ across the genome remains unclear (table 1).

**Table 1.** Summary of known factors involved in prevention of ectopic centromere formation.

factors		function	refs
ubiquitin-mediated proteolysis pathway		control CENP-A level at post-translational level	[44,117–126,128–143]
MBF/E2F, Cdk5rap2		control CENP-A level at transcriptional level	[77,151,152,162]
chromatin remodeling complexes	FACT	facilitate the interaction of E3 ligase with nucleosomal CENP-A	[102,172]
	HIRA	regulation of ubiquitin-mediated CENP-A degradation	[178–180]
	SWI/SNF	destabilize chromatin that contains CENP-A	[182]
	CHRAC	prevent misincorporation of CENP-A at damage sites	[183]
Ccp1		antagonize CENP-A loading at both centromeres and non-centromeric regions	[186]
H2A.Z		inhibit the efficient association of Scm3 with chromatin	[186,189,190]
CENP-C		prevent mistargeting of CENP-A to ectopic sites	[192]

## 9. Concluding remarks and future perspectives

Recent exciting findings have provided substantial insights into mechanisms inhibiting the formation of ectopic centromeres. Many important questions remain unanswered. One of the key questions is how factors involved in prevention of CENP-A mislocalization specifically recognize misincorporated CENP-A? It has been proposed that CENP-A-containing chromatin may be ‘licensed’ by additional modifications that may provide specificity for kinetochore assembly [49]. Further study is needed to shed light on this important issue. It will be also important to understand how different pathways and factors identified in ectopic CENP-A prevention work together to guard the integrity of non-centromeric regions. Furthermore, are there any other ways to prevent ectopic centromere formation? For example, there might be a mechanism to prevent the assembly of the downstream CCAN complex on ectopic CENP-A chromatin. The last few decades have witnessed an explosion of studies focused on the role of CENP-A in cancer and developmental diseases [4,9,10,106–113]. However, how misincorporation of CENP-A can mechanistically cause human diseases, such as cancer, remains elusive. Whether the factors identified above can serve as therapeutic targets in the treatment of cancer is another crucial question. Answering such questions will further advance our fundamental understanding of chromatin organization, and might lead to novel diagnostic and therapeutic avenues in the treatment of human disease resulting from centromere dysfunction.

## Data accessibility

This article has no additional data.
